# Is flow cytometry better in counting malaria pigment-containing leukocytes compared to microscopy?

**DOI:** 10.1186/1475-2875-8-255

**Published:** 2009-11-16

**Authors:** Thomas Hänscheid, Rosangela Frita, Matthias Längin, Peter G Kremsner, Martin P Grobusch

**Affiliations:** 1 Medical Research Unit, Hôpital Albert Schweitzer, Lambaréné, Gabon; 2 Institute of Molecular Medicine, Faculty of Medicine, Lisbon, Portugal; 3 Institute of Tropical Medicine, University of Tübingen, Tübingen, Germany; 4 Infectious Diseases Unit, Division of Clinical Microbiology and Infectious Diseases, National Health Laboratory Service and School of Pathology, Faculty of Health Sciences, University of the Witwatersrand, 7 York Road, Parktown, 2193 Johannesburg, South Africa

## Abstract

**Background:**

Detection of malaria pigment (or haemozoin; Hz)-containing leukocytes may have prognostic relevance in malaria; however, studies reported conflicting results, with microscopic counts suggestive of being inaccurate and imprecise.

**Methods:**

Numbers of Hz-containing leukocytes from a malaria patient obtained with a flow cytometer counting 50.000 gated events were compared with thin film microscopy as applied under field conditions.

**Results:**

Flow cytometry identified 5.8% Hz-containing monocytes and 1.8% Hz-containing neutrophils. The microscopic examination yielded 10% and 13% of Hz-containing monocytes, as well as 0% and 0.5% of Hz-containing neutrophils for observers one and two, respectively.

**Conclusion:**

Novel, robust and affordable cytometric methods should be evaluated in the field as they may assist in utilizing Hz-containing cells as clinically useful parameter.

## Background

Malaria continues to be a major health threat to people living in endemic areas, and reliable early identification of patients at particular risk for progressing towards severe disease remains a challenge. During haemoglobin digestion, malaria parasites detoxify the remaining haem into the crystalline malaria pigment, haemozoin (Hz). When the parasites are liberated into the blood stream, the Hz crystal(s) are also released and removed from the blood by either resident macrophages; or monocytes and granulocytes in the blood. Thus, the presence of Hz-containing leukocytes in the circulation is an indicator of the presence of *Plasmodium *spp. However, pigmented monocytes remain in the circulation for a prolonged period of time and may also indicate a recent, successfully treated malaria episode [1,2]. Moreover, and particularly so in young children with malaria, accurate and precise determination of Hz-containing leukocytes may serve as a prognostic marker for disease severity and progression [3,4].

Hz has interesting physical properties; it is bi-refringent (depolarizing) and paramagnetic. The fact that Hz can be easily detected using dark field or polarizing microscopy led to some interest in the diagnostic value of pigmented leukocytes. The chance finding that one type of automated haematology analyzers (Cell-Dyn^®^, Abbott, Santa Clara, California) also detects Hz-containing leukocytes during routine full-blood counts has revived this interest and led to a series of studies in endemic and non-endemic areas, as reviewed elsewhere [5].

However, whereas even the finding of a single pigmented leukocyte is highly indicative of malaria, the question arose if the number of Hz-containing leukocytes may harbour useful clinical information. In fact, over the last 20 years a couple of studies reported the quantitative determination of these pigmented leukocytes. Most studies established a highly significant, positive correlation with disease severity although the results from different study sites were highly variable [1,6-9], despite the fact that light microscopy, using Giemsa-stained smears or thick films, was common to all studies. The methodology how the pigmented leukocytes were counted differed largely, as has been pointed out recently [4].

However, many of these studies included a rather small number of patients with severe malaria and even less so fatal cases. A recent study circumvented this problem by pooling the data from six different centers from across Africa [10]. This study included an impressive 26,000 children, which lends power to the authors' conclusion that 'pigmented cells are no useful predictor for disease outcome across Africa'. However, a significant methodological limitation merits further consideration. The pigmented leukocytes were counted in thick films [11]: (i) the number of pigmented monocytes was counted in a total of 200 mononucleated cells; and (ii) the number of Hz-containing granulocytes was obtained by counting them in a total of 200 granulocytes. The median value for pigmented granulocytes was 2%, while for pigmented mononuclear leukocytes it was 4%. However, the most striking result is the rather low percentage of patients who had detec4 pigmented mononuclear leukocytes (63%) and granulocytes (37%), as well as the highly significantly discrepant results between the study sites. There were 89% of patients from Libreville, 81% from Lambaréné, 70% from Banjul, 67% from Kilifi, 54% from Blantyre and 50% from Kumasi with pigmented mononuclear leukocytes; there were 70% of patients from Libreville, 55% from Lambaréné, 42% from Kilifi, 40% from Kumasi, 30% from Banjul and 16% from Blantyre with pigmented granulocytes. The odds ratios measuring the associations between pigmented granulocytes and mortality via logistic regression were also very different between the six sites across Africa. The most robust statistical associations were between increased pigmented granulocytes (> 5%) and fatal outcome, which was significant across all sites when assessed by crude odds ratios of 13.6 for Lambaréné, 12.0 for Blantyre, 3.0 for Kumasi, 2.8 for Banjul, 2.8 for Libreville and 1.6 for Kilifi. Adjusted odds ratios maintained this association in Blantyre, Kumasi and Lambaréné [10].

The problem of determining the accurate number of rare cells by microscopy has an equivalent in hematology: the rather imprecise and inaccurate manual 100 white blood cell (WBC) differential count [12]. Basophils or eosinophils are usually found only a few times during such a 100 WBC differential count. For example, a result of 5% eosinophils means that only 5 cells of this characteristic were found while observing 100 WBC. The 95% confidence interval (CI) for this 5% value has been calculated to range from 1% to 12% [12]. In fact, it is this limitation, which led to the widespread use of automated haematology analyzers. These instruments analyse around 10,000 cells for a WBC differential, thus reducing the confidence interval to values within decimals of the measured/detected number of cells; for example, analysing 10,000 cells for the 5% eosinophil value yields a CI from 4.6% to 5.4%.

**Figure 1:**
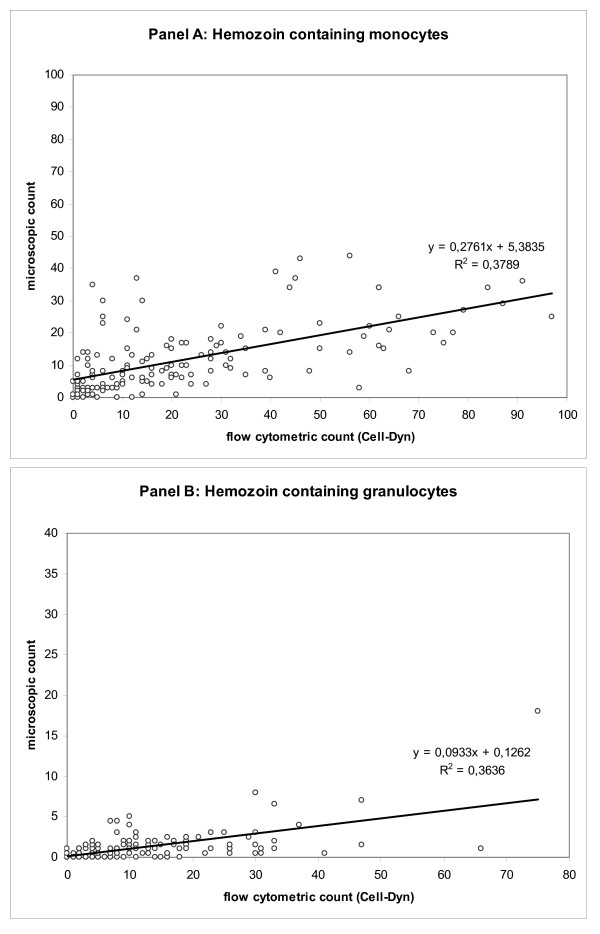
**Comparison of flow cytometric and microscopic determination of Hz-containing leukocytes (panel A, monocytes; panel B, granulocytes)**. 152 samples from malarious patients were used as part of a study on flow cytometric detection of Hz-containing leukocytes [5]. Hz-containing leukocytes were determined by counting 100 monocytes and 200 granulocytes in a thick film, and by image analysis of screenshot from a Cell-Dyn^® ^haematology analyser, as described elsewhere [5]. To allow better scaling in panel A, four outliers were excluded with 173/30, 167/53, 128/33 and 111/50 flow cytometric/microscopic monocyte counts, respectively.

Thus, it is not too surprising that, contrary to the mentioned reports, studies that used flow cytometric methods reported the detection of pigmented leukocytes in most, if not all malaria patients. The various Cell-Dyn^® ^analysers showed a sensitivity for the diagnosis of malaria of 90-95% in malaria-endemic regions, including several African countries [4,13,14]. This means that 90-95% of all individuals with malaria *did *have pigmented monocytes in the peripheral blood that were detectable - a result much higher than in reported studies using microscopy. Moreover, a large number, if not the majority of the patients in the Cell-Dyn^® ^studies had only non-severe malaria; and thus were likely to have considerably lower numbers of pigmented leukocytes. These results are further corroborated by a study that employed image analysis of the Cell-Dyn^® ^on-screen results [4], which showed that 100% of severe malaria cases had pigmented monocytes and still 94% of the non-severe malaria cases; while 97% of severe malaria cases had pigmented granulocytes and 81% of the non-severe cases. It should be noted that the instrument analysed a mean of 1,364 monocytes and 4,175 granulocytes per sample (n = 152), as compared with only 200 mononucleated cells (including lymphocytes) and 200 granulocytes, as reported in the large multicenter study that used microscopy [10]. Furthermore, during this study, Hz-containing monocytes and Hz-containing granulocytes were determined by the Cell-Dyn^® ^instrument and by manual microscopy count of thick films [4]. Overall, a clear tendency becomes apparent. The total number of Hz-containing cells is underestimated by manual microscopy, especially at higher numbers as determined by flow cytometry (Figure 1). Data interpretation should take into account that the Cell-Dyn^® ^instrument's on-screen results constitute a rather approximate representation of cells analysed. Factors like low screen resolution and representation algorithms may cause a data reduction by a factor of around 5 [4]. Thus, the true flow cytometric count is likely to be several times higher than presented in the figures, increasing the difference even more.

## Methods

Further support for this notion was lent by a simple comparison of a flow cytometric count of Hz-containing leukocytes with thin film microscopy. Leukocytes from a patient with *P. falciparum *malaria (0.2% parasitaemia), were labelled with CD14 (monocytes) and with CD16 (granulocytes) (Becton Dickinson, Franklin Lakes, NJ, USA) and erythrocytes were lysed using a Lysis kit (Partec, Münster, Germany). 50,000 gated events were acquired with a flow cytometer (Cyflow^®^, Partec, Münster, Germany) and Hz containing leukocytes determined by measurement of Side-Scatter-Depolarization, as described for the Cell-Dyn^® ^instruments [4]. During microscopy, Hz-containing monocytes were independently counted by two observers as part of a 200-mononuclear-cell count; and Hz-containing neutrophils were counted as part of a 200-polymorph-nuclear cell count, as described recently [10]. Microscopic and flow cytometric counts were then corrected using the Full-Blood-Count result.

## Results and Discussion

Figure 1 depicts the comparison of the flow cytometric and the microscopic determination of Hz-containing leukocytes. Flow cytometry identified 5.8% Hz-containing monocytes and 1.8% Hz-containing neutrophils. The microscopic examination yielded 10% and 13% of Hz-containing monocytes, as well as 0% and 0.5% of Hz-containing neutrophils for observers one and two, respectively.

These results indicate that microscopic counting of Hz-containing cells is prone to large errors. So, if there is an association of Hz-containing leukocytes and severity of malaria, is there any (clinical) point in counting them? It seems that counting them by microscopy may not yield sufficiently accurate and precise results to turn the number of pigmented leukocytes into a clinically useful marker. However, as flow cytometric methods show, it appears too early to dismiss Hz-containing leukocytes as potentially useful clinical markers of malaria severity. The question is: are there any other, less complicated and expensive methods than flow cytometry to determine these cells with higher accuracy than light-microscopy? Recently a prototype of a low-cost image cytometer has been described that may offer one possible answer to this question [15]. It is currently being developed for detection of *Mycobacterium *spp. and *Plasmodium *spp.; and it seems likely that it may also facilitate determination of Hz-containing leukocytes. Whereas rapid diagnostic tests (RDTs) for malaria are of utmost value to assist with establishing a diagnosis of malaria, simple, easily maintainable and affordable flow cytometers adapted to the needs and conditions of rural/semi-rural settings in the South could play an important role in swiftly assisting to predict outcome in malaria, in combination with other much-needed applications in full blood counting, CD4+ counting in HIV/AIDS patients and also TB diagnosis [16,17].

## Conclusion

In conclusion, robust and affordable, purpose-tailored 'no frills' flow cytometric field applications should be tested in the field for diagnostic accuracy and cost effectiveness in comparison with light microscopy as they may assist in overcoming practical limitations of microscopy otherwise so far indispensable for malaria diagnosis.

## Competing interests

The authors declare that they have no competing interests.

## Authors' contributions

TH conceived and designed the study, contributed to the experiments and to data analysis and drafted the paper. RF contributed to the experiments, data analysis and the writing of the paper. ML contributed to the underlying experiments, data interpretation and the writing of the paper. PGK contributed to the design of the study, the data analysis and the writing of the paper. MPG contributed to the design of the study, the data analysis and the writing of the paper. All authors read and approved the final manuscript.
